# Zoonotic Larval Cestode Infections: Neglected, Neglected Tropical Diseases?

**DOI:** 10.1371/journal.pntd.0000319

**Published:** 2009-02-24

**Authors:** Christine M. Budke, A. Clinton White, Hector H. Garcia

**Affiliations:** 1 Department of Veterinary Integrative Biosciences, College of Veterinary Medicine and Biomedical Sciences, Texas A&M University, College Station, Texas, United States of America; 2 Infectious Disease Division, Department of Internal Medicine, University of Texas Medical Branch, Galveston, Texas, United States of America; 3 Department of Microbiology, Universidad Peruana Cayetano Heredia, Lima, Peru; 4 Cysticercosis Unit, Instituto de Ciencias Neurológicas, Lima, Peru; University of Oklahoma Health Sciences Center, United States of America

Recent efforts, including the launching of *PLoS Neglected Tropical Diseases*, have begun to bring attention to conditions now broadly referred to as neglected tropical diseases (NTDs). Many of these diseases, which affect the socioeconomically disadvantaged of the world, had not been thoroughly examined and included in large-scale burden assessment undertakings, such as the Global Burden of Disease Study, and had not been prioritized at a regional or global level [1]. Recently, global partnerships have been developed that are pushing for inclusion of NTDs in infectious disease research and disease control initiatives. However, an important group of disease-causing organisms has often been excluded, namely the zoonotic larval cestodes [2].

Echinococcosis, attributable to the larval stage of *Echinococcus* spp., and cysticercosis, caused by larval *Taenia solium*, are two such parasitic conditions. They are both widely distributed throughout the world. Both disproportionately affect the socioeconomically disadvantaged segments of society and cause serious human morbidity due to the effect of the parasite larvae in the liver, lung, or brain of the host. Both have also caused collateral economic damages to poor farmers because of infection of their animals, which are no longer as marketable.

The most important zoonotic *Echinococcus* species, from a public health standpoint, are *E. granulosus* and related species that cause cystic echinococcosis (CE), and *E. multilocularis*, the cause of alveolar echinococcosis. Humans are infected by ingesting eggs shed in the feces of canine definitive hosts. The result is the formation of slow-growing larval cysts in the liver, lungs, or other organ systems, which eventually produce clinical signs from mass effects, allergic reactions, or through tissue necrosis/fibrosis [3]. In contrast, humans act as the definitive host for *Taenia solium* infection, with the adult worms residing in the intestinal tract. Swine become infected upon ingestion of eggs shed in the feces of an infected human tapeworm carrier resulting in larval cyst formation (cysticercosis) in muscle and other tissues. The cycle continues when humans acquire the tapeworm form by ingesting undercooked infected pork. Humans can also become infected with the larval stage of this parasite via the ingestion of infective eggs. When cyst formation occurs in the brain, the resulting condition is termed neurocysticercosis (NCC), a major cause of seizures and epilepsy in *T. solium*–endemic regions [4],[5].

Only recently, the World Health Organization included echinococcosis and cysticercosis as part of a Neglected Zoonosis subgroup for its 2008–2015 strategic plan for the control of NTDs [6],[7]. Both echinococcosis and cysticercosis are also to be included in a review of the Global Burden of Disease Study, which is currently underway. However, even with these steps forward, there are limited data with which to estimate the global burden of disease from larval cestodes. In contrast to many of the NTDs, which can be readily identified by obvious clinical lesions (e.g., filariasis, Buruli ulcer) or a simple laboratory test (e.g., stool examination for geohelminths), diagnosis of larval cestode infections typically requires a high index of suspicion and access to confirmatory imaging studies. For example, initial diagnosis of CE typically requires imaging studies such as abdominal ultrasound or chest X ray [8]. These techniques have only recently been moved into field studies. However, when imaging studies have been applied in endemic areas, the burden of disease is quite high [9],[10]. While CE is not, strictly speaking, a tropical disease, it does result in a high disease burden in under-developed regions of the world, including areas of North Africa, the Middle East, Central Asia, and China. Preliminary estimates of the global burden of CE, in terms of disability-adjusted life years (DALYs) lost, suggest that CE may cause disability on at least a similar level to numerous better-known NTDs, including Chagas disease, dengue, onchocerciasis, and trypanosomiasis [11]. However, even this estimate is likely conservative since it fails to account for the burden of unrecognized disease.

NCC, which is due to *T. solium*, is also widely distributed, with endemic regions in Mexico, Central and South America, and parts of Africa and Asia [4]. It causes a wide range of neurologic symptoms that can be confused with other conditions. Diagnosis typically requires access to computer tomography (CT) or magnetic resonance imaging (MRI) scans. When these techniques have been applied in endemic areas, NCC has been recognized as the major cause of seizure disorders as well as an important cause of hydrocephalus and stroke [12],[13]. In some field studies, it caused a substantial proportion of all cases of epilepsy. The number of DALYs lost globally due to *T. solium* has not yet been determined, but is most likely substantial [14]–[16]. In addition to human health losses, both of these conditions also result in considerable livestock-associated production losses due to cyst formation in the intermediate hosts [11],[15].

The question then arises as to why these parasitic diseases, obviously important from both a human health and a livestock production perspective, are still not receiving the attention they are due. One possibility is the difficulty in diagnosis. While the advanced diagnostic techniques required to diagnose these parasites are becoming more prevalent throughout the world, the populations most at risk still have limited physical and financial access to these imaging modalities. For example, information is known about CE in nomadic Turkana populations of Kenya only because a small group of dedicated researchers has been undertaking community ultrasound studies in these villages for over two decades [17]. However, CE extends far beyond the borders of these hyperendemic villages, with limited ability for early diagnosis. In addition, without a definitive image-based diagnosis, many of the clinical manifestations of NCC, including epilepsy, acute symptomatic seizures, severe headaches, and psychiatric disturbances, are not attributed to larval cestode infection. As a result, both conditions are vastly under-reported in the regions where they have the largest impact.

Another reason for the zoonotic larval cestodes' neglected status may be the chronic nature of these conditions [18]. Clinical signs associated with echinococcosis can take five to ten years to develop, with clinical signs associated with NCC also taking months or years to appear. For government administrations dealing with a myriad of public health crises, this lack of acute symptoms may make these conditions seem less dire than those with a shorter incubation period. In addition, unlike diseases with acute outbreaks that result in national and international media coverage, echinococcosis and NCC are rarely, if ever, publicized. As a result, there is a lack of outcry from the populations of endemic regions to pressure local governments to implement additional control efforts. Since both conditions also affect livestock, it is possible that the perception of the public health sector, in many endemic regions, is that these diseases are an agricultural problem and are, therefore, primarily the responsibility of the agricultural/veterinary sector. As with human disease, livestock disease is also slow and insidious, with many farmers not recognizing that they have a problem.

A third potential reason for the neglected status of both echinococcosis and cysticercosis is that treatment is difficult [18]. Treatment for both diseases can include surgical resection of cysts (this is especially common for echinococcosis), treatment with an anthelminthic agent (which may be prolonged and is often only partially effective), and additional supportive therapy, such as anti-inflammatory and anticonvulsant medications for NCC [3],[4]. Finally, control program implementation, for both of these conditions, is a long-term commitment, which may include anthelminthic treatment of definitive hosts, vaccination, treatment and/or inspection of intermediate hosts, and education initiatives [19],[20]. Effective control, therefore, requires cooperation between the medical establishment, public health sector, and the veterinary/agricultural sector, with communication between these fields often not well established in many developing regions. When proposing control measures for an NTD, a systems approach with other co-endemic NTDs has been suggested as a cost-effective option [7],[21]. This approach may also be applicable to the zoonotic larval cestodes. For example, the integrated control of CE, leishmaniasis, and rabies has been proposed for co-endemic regions of North Africa, and a combined approach towards echinococcosis, rabies, and tuberculosis control has been suggested for regions of East Africa and China [18]. Cysticercosis is also likely to be a good candidate for an integrated approach to control, such as in areas co-endemic with schistosomiasis.

An increased focus on burden assessments related to zoonotic larval cestodes should increase the profile of these important conditions. However, until the full socioeconomic impact of echinococcosis and cysticercosis is better defined, and an improved linking of the public health and agricultural/veterinary sectors is achieved, we believe that zoonotic larval cestodes will continue to be treated like neglected stepchildren, even among the NTDs. If the goal of recent NTD endeavors is to truly address those conditions that significantly impact the world's most disadvantaged populations, greater attention needs to be directed at zoonotic larval cestode infections, especially given their impact on both human health and livestock production, their difficulty in diagnosis, their chronic nature, and the complexity of treatment and control.
